# Worldwide cohort study of 46, XY differences/disorders of sex development genetic diagnoses: geographic and ethnic differences in variants

**DOI:** 10.3389/fgene.2024.1387598

**Published:** 2024-06-10

**Authors:** Chen Jiali, Peng Huifang, Jiang Yuqing, Zeng Xiantao, Jiang Hongwei

**Affiliations:** ^1^ Henan Key Laboratory of Rare Diseases, The First Affiliated Hospital, and College of Clinical Medicine of Henan University of Science and Technology, Luoyang, China; ^2^ Center for Evidence-Based and Translational Medicine, Zhongnan Hospital of Wuhan University, Wuhan, China

**Keywords:** 46, XY DSD, genetic diagnosis, worldwide cohort, geographic and ethnic differences, diagnostic method, multiple mutations

## Abstract

Differences/disorders of sex development (DSDs) in individuals with a 46, XY karyotype are a group of congenital disorders that manifest as male gonadal hypoplasia or abnormalities of the external genitalia. Approximately 50% of patients with 46, XY DSDs cannot obtain a molecular diagnosis. The aims of this paper were to review the most common causative genes and rare genes in patients with 46, XY DSDs, analyze global molecular diagnostic cohorts for the prevalence and geographic distribution of causative genes, and identify the factors affecting cohort detection results. Although the spectrum of genetic variants varies across regions and the severity of the clinical phenotype varies across patients, next-generation sequencing (NGS), the most commonly used detection method, can still reveal genetic variants and aid in diagnosis. A comparison of the detection rates of various sequencing modalities revealed that whole-exome sequencing (WES) facilitates a greater rate of molecular diagnosis of the disease than panel sequencing. Whole-genome sequencing (WGS), third-generation sequencing, and algorithm advancements will contribute to the improvement of detection efficiency. The most commonly mutated genes associated with androgen synthesis and action are *AR*, *SR5A2*, and *HSD17B3*, and the most commonly mutated genes involved in gonadal formation are *NR5A1* and *MAP3K1.* Detection results are affected by differences in enrollment criteria and sequencing technologies.

## Introduction

Sex differentiation in humans is a delicate process regulated by multiple genes and is divided into three stages: the bipotential gonad stage, sex determination, and gonadal differentiation. In the bipotential gonad stage, which lasts from fertilization to the sixth week of embryo formation, there are no significant morphological differences between female (XX) and male (XY) embryos. In the sex determination stage, the successful expression of the *SRY* gene on the Y chromosome in XY embryos causes a complex cascade of expression of dozens of genes (*SOX9*, *SF1*, *CBX2*, *WT1*, *FOG2*, *GATA4*, and other genes) in the testicular pathway (Sekido, 2010). In contrast to testicular determination, the absence of the *SRY* gene in an XX embryo coincides with the expression of pro-ovarian genes (*RSPO1* and *FOXL2*) that dictate ovarian fate. During the gonadal differentiation stage, in XY embryos, Sertoli cells secrete anti-Müllerian hormone (AMH) to induce Müllerian duct regression, and Leydig cells secrete androgens to induce the Wolffian duct to differentiate into the paratestis, vas deferens, and seminal vesicles. Due to the absence of AMH and androgen, the Müllerian duct of XX embryos differentiates into the oviduct, uterus, and upper vagina. In all of these processes, molecular-level changes can lead to differences/disorders of sex development (DSDs), which result in discordance between an individual’s sex chromosomes, gonads, and/or anatomic sex (Hughes et al., 2006). DSDs are classified into three subtypes (sex chromosome DSDs, 46, XY DSDs, and 46, XX DSDs) according to the sex chromosomes; these subtypes are widely recognized internationally (Hughes et al., 2006). The clinical presentations of DSDs are complex and varied. Sex chromosome DSDs account for 15% of all DSDs, including Klinefelter syndrome (47, XXY), Turner syndrome variants with Y chromosome contributions (typically mosaic 45, X/46, XY, with or without isodicentric Y), segmental deletions of the X chromosome, or translocations of an SRY-containing Y fragment to the X chromosome (Délot and Vilain, 2021a).

Among 46, XX individuals with genital anomalies, 90%–95% of cases are associated with congenital adrenal hyperplasia (CAH) caused by *CYP21A2* deficiency, and patients may present with varying degrees of external genital masculinization in infancy and early childhood and primary amenorrhea or infertility in adolescence or adulthood (Speiser et al., 2018). Some patients with DSDs possess a 46, XY chromosome complement and can present with variable degrees of virilization of their external genitalia, from a small penis or mild hypospadias to full female external genitalia. The 46, XY DSD subtype can be further subdivided into 46, XY gonadal dysgenesis (GD), disorders of androgen synthesis or action, and disorders of AMH synthesis or action. Gonadal development disorders include complete gonadal hypoplasia (previously known as Swyer syndrome), partial gonadal hypoplasia, gonadal degeneration, and ovotesticular DSD (King and Conway, 2014). The external masculinization score (EMS) is an objective and standardized tool for describing the external genitalia of male infants (Ahmed et al., 2000). The EMS is calculated from the following features: micropenis (0/3), fused scrotum (3/0), position of the urethral opening (3/2/1/0), and position of the right and left gonads (1.5/1.0/0.5/0). Regardless of its phenotypic variability, the etiology of 46, XY DSDs is ultimately impaired steroid synthesis or action or a defective/affected gonadal differentiation process.

Currently, there are few investigations of DSDs worldwide. To generalize the prevalence and geographic variability of 46, XY DSD pathogenic genes, we collected data from worldwide cohorts to identify patterns.

### Diagnosis of DSDs

The incidence of newborns with ambiguous genitalia ranges from 1/5,000 to 1/4,500 (Lee et al., 2006; Thyen et al., 2006). Because patients with DSDs always have a complex etiology and clinically distinct phenotypes of abnormal internal or external genital development, overlapping phenotypes and genetic heterogeneity make diagnosis challenging (Délot and Vilain, 2021b). Currently, the diagnosis of DSDs relies on analyses complementary to physical examination, such as sex hormone testing, imaging, and molecular testing (Délot and Vilain, 2021b). Hormone tests for diagnosis and hormone replacement therapy require blood or urine samples, which can be used to analyze disorders of a part of the gonadal axis. Moreover, ordinary biochemical tests are unable to identify the cause of androgen abnormalities. Prenatal ultrasound (US) can detect signs of DSDs but is often limited by technology and gestational age (Wisniewski et al., 2019). Neonates often present with atypical external genitalia, children with bilateral inguinal hernias, adolescents with abnormal development of secondary sexual characteristics during puberty, and adults with infertility. Ultrasonography can reveal undescended unilateral or bilateral testes and even uterus- and ovary-like tissue. First, sex chromosome karyotyping is performed to determine whether there are abnormalities in the number or structure of sex chromosomes or a difference between genetics and gender identity. The next step is obtaining a molecular diagnosis (Délot and Vilain, 2021b).

Currently, the most utilized technology for diagnosing DSDs is next-generation sequencing (NGS), but many patients do not receive a molecular diagnosis (Baxter et al., 2015b). It is more difficult to obtain a molecular diagnosis for 46, XY DSD patients. Over the past decade, many cohorts have been studied worldwide to determine the cause of DSDs using NGS, but the diagnosis rate for most studies of 46, XY DSD patients does not exceed 50% (Arboleda et al., 2013; Baxter et al., 2015a; Eggers et al., 2016; Kim et al., 2017; Özen et al., 2017; Hughes et al., 2019; Globa et al., 2022; Zidoune et al., 2022). We analyzed common pathogenic mutations and novel genes, as well as possible causative genes, from various cohort studies of DSDs worldwide and observed whether there are geographical and ethnic differences in mutated genes or loci.

### Worldwide cohorts

The cohorts mentioned in this paper were identified by searching PubMed for relevant articles using the keywords “46, XY DSD, next-generation sequencing (NGS), whole-exome sequencing (WES), and genetic diagnosis.” We excluded cohort studies targeting only a certain phenotype or a particular type of disease, such as a cohort that included patients with only hypospadias or androgen insensitivity syndrome (AIS). We categorized the countries where the study was conducted into Asia, Africa, North America, South America, Europe, and Oceania. The aim was to understand the main pathogenic genes of 46, XY DSDs worldwide and observe whether there are geographical and ethnic specificities. Information on the mutated genes detected in each study cohort, the mode of diagnosis, and the diagnosis rate are shown in Table 1.

**TABLE 1:** Worldwide cohorts study for molecular diagnosis of DSD.

Time^a^	Country	Number of 46, XY DSD patients	Method	Diagnosis rate (%)^b^	Common genes mutations^c^	Other genes mutations and candidate variants^d^
2013	America (Arboleda et al., 2013)	5	MPS	—	—	—
2015	America (Baxter et al., 2015a)	40	WES	35.0	*MAP3K1, AMHR2, HSD17B3, CHD7*	*DHH, FGFR1, LHCGR, MAMLD1, NRP1, WT1*
2016	Australia (Eggers et al., 2016)	278/326	MPS	42.4	*AR, NR5A1, SRD5A2*	*AMH, AMHR2, CHD7, CREBBP, DHH, FGF8, FGFR1, FGFR2, GATA4, GNRHR, HSD17B3, HSD3B2, KAL1, LHCGR, MAMLD1, MAP3K1, POR, PROK2, PROKR2, SOX9, SRY, STAR, WDR11, WT1, ZFPM2*
2016	China (Dong et al., 2016)	13	Panel	69.23	*AR*	*CHD7, CYP17A1, NR0B1, SRD5A2, SRY*
2017	Korea (Kim et al., 2017)	37/44	Panel	24.3	*AR, CYP17A1*	*SRD5A1, ATRX, MAP3K1 DMRT1/2*
2017	Turkey (Özen et al., 2017)	20	Panel	45.0	*HSD17B3*	*LHCGR, SRY, WT1*
2018	China (Wang et al., 2018)	70	Panel	42.86	*AR, NR5A1, SRD5A2*	*BMP4, ESR1, LHCGR, MAMLD1, SOX3, SRY, WT1*
2019	The United Kingdom (Hughes et al., 2019)	73/80	Panel	34.2	*AR, HSD17B3, SRD5A2*	*AMH, AMHR2, AR, DHCR7, HSD17B3, HSD3B2, LHCGR, MAMLD1, NR5A1, SRD5A2, WT1*
2021	China (Xia et al., 2021)	9	Panel	—	*AR, NR5A1, SRY*	*LHCGR*
2022	Ukraine (Globa et al., 2022)	71/79	WES	46.5	*AR, NR5A1*	*AMHR2, ANOS1, CACNA1A, DHX37, FGFR11, GATA4, GLI2, HSD17B3, MYRF, SRD5A1, TBCE, WT1*
2022	China (Xie et al., 2022)	74	Panel+WES	41.9	*AR, CYP17A1, FGFA1*	*AKR1C2, AMH, BBS1, BBS12, CDKN1C, CYP11A1, DHCR7, ESR1, FRAS1, GLI3, HSD3B2, LHCGR, LISS1R, MYPF, NR5A1, NROB1, PCNT, PMM2, POR, SOS1, SRD5A2, SRY, STAR, WDR11, WDR35, ZFPM2*
2021	Algeria (Zidoune et al., 2022)	122/125	WES	49.6	*AR, HSD17B3, NR5A1, SRD5A2*	*ANOS1, BBS7, CCDC141, CHD7, DUSP6, FEZF1, FGFR1, FGFR3, FLNA, FLRT3, FSHB, GNRHR, GPC3, HOXA13, HS6ST1, KAT6B, LHCGR, LHX3, LHX4, NR5A1, NRAS, OFD1, PLXNA3, PRKAR1A, PROK2, PROKR, PROKR2, PROP1, RNF216, RXFP2, SEMA3A, SEMA3F, SHH, SPRY4, ZFPM2*
2022	Brazil (Gomes et al., 2022)	209	MPS/panel	59.3	*AR, SRD5A2, NR5A1*	*AMH, AMHR2, CBX2.2, CHD7, CYP17A1, DHH, DHX37, ESR2, FGFR2, GATA4, HSD17B3, HSD3B2, LHCGR, MAP3K1, SRY, WT1, ZFPM2*
2023	China (Zhang et al., 2023)	70	WES	64.3	*AR, SRD5A2, NR5A1*	*DHX37, GATA4, HSD17B3, MYRF, SRY, PPP2R3C*

^a^ The year indicated is when the article was published.

^b^ The diagnostic rate was calculated as the percentage of P/LP variants detected at the time of the cohort study.

^c^ The common gene mutations mentioned in the Table 1 are the top three (or tied) mutation rates in each study.

^d^ Other mutated genes are sorted in alphabetical ascending order.

### Asia

#### China

Studies on the molecular detection of 46, XY DSD patients in China included a total of six cohorts. A study published in 2016 by Southwest Hospital, Third Military Medical University, Chongqing, China, included 21 Han Chinese DSD patients: 13 patients with 46, XY DSDs and 8 patients with 46, XX DSDs, and no clear inclusion criteria were described (Dong et al., 2016). A total of 9 (69.23%, 9/13) of the 13 patients with 46, XY DSDs received a molecular genetic diagnosis. NGS-based targeted sequencing technology was used for the detection of comprehensive DSD-related genes (a total of 2,972 exons and 1,078,042 bases of 219 genes). Mutations in the *AR* gene, which was the most common pathogenic gene in this cohort, were detected in five (55.56%) of nine probands. The other mutations detected were located in *SRD5A2* (22.22%, 2/9), *NR0B1* (22.22%, 2/9), *CYP17A1* (11.11%, 1/9), *GK* (11.11%, 1/9), and *SRY* (11.1%, 1/9) (Dong et al., 2016). The detected mutations were limited to a few common DSD genes due to the small sample size of this study, but this study also provides new evidence for the molecular diagnosis of DSDs and supports subsequent studies.

Although the cohort was small and the paper did not provide clear inclusion criteria, the molecular diagnostic results indicated that *AR* mutations were most common in this cohort. In contrast, the Department of Endocrinology of the Ninth People’s Hospital of Shanghai Jiao Tong University School of Medicine published another study of 46, XY DSD patients in 2018 (Wang et al., 2018); patients with classical complete AIS (CAIS) were excluded, and *AR* mutations remained in the top three in this cohort (Wang et al., 2018). A study in Shanghai, China, excluded patients with CAIS, CAH, or syndromic DSD and those who underwent virilization or sex changes after puberty. They recruited 70 patients with 46, XY DSDs who could not be diagnosed on the basis of typical clinical presentation and conventional candidate genes (including 33 DSD candidate genes and 47 potential disease-causing genes). The final diagnostic rate obtained by panel sequencing was 42.7% (30/70). Patients with micropenis, cryptorchidism, and hypospadias had the highest diagnosis rate (58%). The most frequently mutated gene was *SRD5A2* (15.04%, 17/113), followed by *NR5A1* (8.84%, 10/113) and *AR* (7.96%, 9/113). Even after excluding patients with CAIS, the probability of an *AR* mutation was still high, which demonstrates the importance of *AR* mutations in the common 46, XY DSD etiology and in sexual development. Clinically, female patients often have primary amenorrhea in adolescence or insufficient masculinization of male internal and external genitalia. Among 9 patients with 46, XY DSDs whose gender identity was female, studied by Xia et al. (2021), more than half (55.6%) of the adolescent or adult patients had primary amenorrhea, 3 had external genital abnormalities, and 2 had masses in the bilateral inguinal region. The molecular diagnoses of the three patients with external genital anomalies were the *NR5A1* mutations c.937C>T and c.398delC and *LHCGR* mutation, c.265A>T from the father and c.422T>C from the mother (Xia et al., 2021). Because this cohort of women with 46, XY DSDs was not randomized, they were not included in this analysis. However, this study still reveals that girls with primary amenorrhea should be aware of the possibility of 46, XY sex chromosomes.

In two studies of 46, XY DSD patients from Peking Union Medical College in Beijing, China, the most common mutated genes were *AR*, *NR5A1*, and *SRD5A2* (Yu et al., 2021; Zhang et al., 2023). The two studies had the same inclusion criteria: patients with a sex chromosome karyotype of 46, XY and an external genital malformation. The most common pathogenic genes in both cohorts were *SRD5A2*, *NR5A1*, and *AR* (Yu et al., 2021; Zhang et al., 2023). The first article was published in 2021, and a total of 87 patients were eventually enrolled in the study. The final diagnosis rate was 42.5% by panel NGS (including 32 reported 46, XY DSD pathogenic genes and 51 genes related to gonadal development or differentiation). Patients with *CYP17A1* mutations who presented with abnormal gonadal development combined with hypertension and hypokalemia were excluded. However, *CYP17A1* mutations were still found in three patients in this cohort (Yu et al., 2021). The second article was published in 2023 and included 70 patients in total. A molecular diagnosis was identified in 64.3% (45/70) of patients in this cohort. The higher diagnosis rate in this cohort compared to other Chinese cohorts may be because this cohort had more patients with androgen synthesis or action and more severe clinical phenotypes, and the patients were sequenced using WES technology (Zhang et al., 2023).

In a 2022 study from Guangzhou, China, the overall diagnostic rate using a panel (108 candidate genes) or WES was 41.9% (31/74) in 74 patients with 46, XY DSDs (Xie et al., 2022). The diagnostic rates were 43.6% (24/55) for panel sequencing and 40.9% (9/22) for WES; in total, 52 patients were sequenced by panels alone, 19 patients by WES alone, and 3 patients with both panels and WES. Variants associated with steroid hormone synthesis and activation, such as *AR* (4.05%), *CYP17A1* (4.05%), and *FGFR1* (4.05%), were the most common in this cohort (Xie et al., 2022). There was little difference in the number of gene mutations detected in this cohort.

#### Korea

After excluding patients with sex chromosome abnormalities and 46, XX DSDs with SRY (+), a Korean cohort included 37 patients with 46, XY DSDs and 7 patients with 46, XX DSDs (Kim et al., 2017). In the 46, XY DSD cohort, pathogenic genes were identified in nine patients through targeted exome sequencing of 67 known DSD-associated genes, and the most common mutated genes were *AR* and *CYP17A1*. Similar to previous studies, a molecular diagnosis in patients with mild phenotypes such as micropenis or isolated hypospadias was difficult to obtain.

#### India

In 2019, MR Nagaraja et al. (2019) investigated the monogenic causes and prevalence of 46, XY DSDs and reported that pathogenic genes causative of AIS, steroid 5 alpha-reductase type 2 deficiency (SRD5A2), and gonadal hypoplasia were most common in India.

## Summary of the Asian cohorts

Patients with severe phenotypes had a higher molecular diagnosis rate, and patients with DSDs with concurrent hypospadias, micropenis, and cryptorchidism had the highest detection rate (58%) (Wang et al., 2018). Similar results were obtained in a molecular study by Zhang et al. (2019) of 130 Han Chinese individuals with varying degrees of hypospadias, with 25 patients receiving a molecular diagnosis. Of the 25 patients who received a molecular diagnosis, patients with hypospadias combined with micropenis had the highest detection rate (68%), followed by patients with combined micropenis and cryptorchidism (28%) and patients with combined cryptorchidism (4%). Patients with isolated hypospadias in this study did not receive a molecular diagnosis.

We found that the rate of positive diagnosis correlated with the severity of the clinical presentation and possibly with the mode of testing and the number of individuals. However, Vuthy E applied next-generation DNA sequencing panel technology to 297 boys with isolated, mainly mild forms of hypospadias (mostly mild hypospadias, no micropenis, and no undescended testis) and obtained surprising results (Ea et al., 2021). Genes involved in the process of steroid synthesis had a higher detection rate in this cohort, including *HSD17B3* (27.78%, 10/36) and *SRD5A2* (11.11%, 4/36). The genes involved in the process of gonadal development detected were *POR* (16.67%, 6/36), *NR5A1* (11.11%, 4/36), and *STAR* (8.33%, 3/36).

The diagnostic rate in this study increased with increasing severity of hypospadias, which is believed to be true for the immediate range (from anterior hypospadias (4.9%) to penoscrotal hypospadias (14.3%)). In this cohort, 5.5% (16/293) of patients with isolated hypospadias were definitively diagnosed (Ea et al., 2021).

The common etiology of 46, XY DSD patients was ultimately attributed to impaired steroid synthesis and action or problems in the gonadal decision differentiation process. Asian cohort studies of patients with 46, XY DSDs have shown *AR*, *NR5A1*, and *SRD5A2* to be the most commonly mutated genes, which is consistent with a common DSD etiology.

Although some cohorts excluded patients with CAIS, *AR* was still the most common gene detected (Wang et al., 2018). The results of these cohort studies suggest *AR* is a common causative gene for DSDs. The *AR* gene is located on the X chromosome and encodes the androgen receptor, also known as the dihydrotestosterone receptor. CAIS is caused by complete androgen insensitivity due to AR inactivation. Even after excluding CAIS patients, the probability of an AR mutation was still the highest. Hundreds of AR mutations, mainly located in the ligand-binding domain, have been reported in CAIS. The majority of AR mutations originated from the mother (approximately 66.67%), while the remaining mutations (approximately 33.33%) arose from somatic and *de novo* mutations (Hiort et al., 1998).

However, in these articles, the demographics of the patients in the cohorts are not clear, and we cannot guarantee that all patients are residents of the city where the hospital is located, except for the cohort of Zhang Wangyu et al., who mentioned that all patients were Han Chinese. Therefore, it is not possible to determine the variability in the regional prevalence of XY DSD patients in China.

### Europe

#### The United Kingdom and Ukraine

A total of 73 patients with 46, XY DSDs were included in the UK study, and the diagnostic rate of 46, XY DSDs by a panel (including 30 genes) was 34.2% (25/73). The most common mutated gene in this study was *AR* (9.59%, 7/73), followed by *HSD17B3* (6.85%, 5/73) and *SRD5A2* (5.48%, 4/73) (Hughes et al., 2019). The Ukrainian study excluded patients with sex chromosomal abnormalities and patients with CAH and included 71 patients with 46, XY DSDs. Using WES, 46.2% of patients were diagnosed. The most common pathogenic genes detected in this study were *AR* (15.49%, 11/71) and *NR5A1* (7.04%, 5/71) (Globa et al., 2022).

#### Turkey

The study in Turkey, which was reported in 2016, excluded patients with mutations in the *AR* and *SRD5A2* genes identified by Sanger sequencing, and a total of 20 patients with 46, XY DSDs were enrolled for panel sequencing. The overall diagnostic rate was 45% (9/20) (Özen et al., 2017). An *HSD17B3* gene mutation was detected in six patients. The remaining patients harbored mutations in the *LHCGR*, *WT1*, and *SRY* genes. The rate of *HSD17B3* mutations in this study may be high because the *AR* and *SRD5A2* mutations were excluded. However, in this cohort, 14 patients had consanguineous parents, and the effect of consanguineous marriage cannot be ruled out (Özen et al., 2017) as the reason for the high rate of *HSD17B3* mutations. The policies on consanguineous marriages vary from country to country, and there is no study cohort with a larger sample of consanguineous marriages worldwide.

### Africa

#### Algeria

The Algerian cohort included 122 Algerian patients with 46, XY DSDs analyzed using WES (patients with suspected or confirmed CAH and sex chromosome abnormalities were excluded), and the overall diagnosis rate was 50.82% (62/122); the diagnosis rate of 46, XY nonsyndromic DSD patients was 42.2% (35/83), and that of 46, XY syndromic DSD patients was 69.2% (27/39). The *AR*, *HSD17B3*, *NR5A1*, and *SRD5A2* genes were the most frequently mutated genes in this cohort (Zidoune et al., 2022).

### North America

#### The United States of America

Previous studies using WES to diagnose 46, XY DSD identified genetic causes in 35% (14/40) of patients and six variants of uncertain significance (VUSs) (15%) (Arboleda et al., 2013). A related study using panel sequencing (including 64 genes) in the United States included 40 XY patients for whom a genetic diagnosis could not be obtained. No commonly mutated genes, such as *AR*, *NR5A1*, or *SRA52*, were detected. WES was also performed on these patients. The *MAP3K1* variant was identified in four patients (Baxter et al., 2015a).

### South America

#### Brazil

Patients with syndromic DSDs, dysmorphic features, developmental delay and/or intellectual disability, and the presence of more than two malformations in addition to genital anomalies were excluded from the São Paulo Hospital, Brazil, study (Gomes et al., 2022). A total of 209 patients were retrospectively included, all of whom had a 46, XY karyotype. Patients were divided according to time, using 2015 as the cutoff; patients before 2015 (168/209) were analyzed using panel testing, and patients after 2015 (41/209) were analyzed using massively parallel signature sequencing (MPSS). The molecular diagnosis rate of patients in the panel group was 59.5% (100/168). Targeted massively parallel sequencing (TMPS) was performed in 59 patients without a diagnosis and in 41 patients after 2015. Ten patients without a molecular diagnosis underwent WES. The most common mutated genes were, in order, *AR* (9.57%, 20/209), *SRD5A2* (7.18%, 15/209), and *NR5A1* (6.70%, 14/209)*.* Five patients in this cohort were found to have polygenic mutations. Three of these patients had an unknown clinical etiology, and three coexisting mutations were found: *WWOX* and *DHX37*, *NR5A1* and *DHX37*, and *MAP3K1* and *GATA4* (Gomes et al., 2022). Two patients with gonadal dysgenesis had missense mutations in two genes, *FGFR2* and *MAP3K1* (Table 2).

**TABLE 2:** Patient has mutations in two or more genes.

Patients origin	Gene	Chr	Variant	Protein alteration	ACMG	Clinical manifestations
Brazil (Gomes et al., 2022)	*WWOX*	chr12	c.253T>G	p.Tyr85Asp	VUS	
	*DHX37*	chr12	c.2209G>A	p.Ala737Thr	VUS	
Brazil (Gomes et al., 2022)	*NR5A1*	chr9	c.77C>T	p.Gly26Val	LP	
	*DHX37*	chr12	c.1474G>C	p.Ala494Pro	VUS	
Brazil (Gomes et al., 2022)	*MAP3K1*	chr5	c.1628A>G	p.His543Arg	VUS	
	*GATA4*	chr8	c.644G>T	p.Arg215Ile	VUS	
Brazil (Gomes et al., 2022)	*FGFR2*	chr10	c.1358C>T	p.Ser453Leu	LP	
	*MAP3K1*	chr5	c.1916T>C	p.Leu639Pro	LP	
China (Wang et al., 2018)	*NR5A1*	chr9	c.634G>A	p.G212S	P	Phenotypic female with complete gonadal dysgenesis, vulvar dysplasia, and, primary amenorrhea, no testis, or ovarian structure
	*SRY*	chrY	C.227G>T	p.R76L	LP	
China (Wang et al., 2018)	*NR5A1*	chr9	c.86C>A	p.T29K	LP	Micropenis, perineal hypospadias, undescended testes and bifid scrotum, FSH and LH ↑, testosterone↓
	*AR*	chrX	c.884T>C	p.L295P	LP	
China (Wang et al., 2018)	*NR5A1*	chr9	c.1109G>A	p.C370Y	LP	Genital dysplasia and gonadal dysgenesis
	*SOX3*	chrX	c.157G>C	p.V53L	LP	
China (Wang et al., 2018)	*NR5A1*	chr9	c.1289G>T	p.S430I	LP	Gonadal dysplasia and relatively normal testosterone level
	*WT1*	chr11	c.19A>C	p.T7P	LP VUS (clinvar)	
China (Wang et al., 2018)	*AR*	chrX	c.1768G>C	p.G590R	LP	
	*BMP4*	chr14	c.806C>T	p.R269Q	LP	
China (Wang et al., 2018)	*SRD5A2*	chr2	c.680G>A	p.R227Q	P	
	*SOX3*	chrX	c.157G>C	p.V53L	LP	
China (Wang et al., 2018)	*ESR1*	chr6	c.433G>A	p.G145S	P	
			c.437C>A	p.P146Q	LP	
China (Dong et al., 2016)	*CYP11A1*	chr15	c.1148A>T	p..E383V	LP	Severe hypospadias, micropenis
	*STAR*	chr8	c.667 T>C	p.V226A	LP	
China (Dong et al., 2016)	*HSD3B2*	chr1	c.13 T>A	C5S	LP	Micropenis
	*AR*	chrX	c.902A> G	K301R	LP	
China (Dong et al., 2016)	*NR5A1*	chr9	c.250C>T	R84C	P	Severe hypospadias, micropenis
	*ZFPM2*	chr8	c.629G>C	S210T	LP	
China (Dong et al., 2016)	*MYRF*	chr11	c.2594C>T	S865L	VUS	Severe hypospadias, bilat cryptorchidism
	*PCNT*	chr21	c.3358C>T	R1120C	VUS	
	*SOS1*	chr2	c.932 T>G	F311C	VUS	
	*FRAS1*	chr4	c.2647G>A	V883M	VUS	
China (Zhang et al., 2023)	*AR*	chrX	c.2200C>G	p.Q734E	VUS	
	*SRD5A2*	chr2	c.16C>T	p.Q6^a^	LP	
			c.578A>G	p.N193S	LP	
Brazil (Gomes et al., 2022)	*WWOX*	chr12	c.253T>G	p.Tyr85Asp	VUS	
	*DHX37*	chr12	c.2209G>A	p.Ala737Thr	VUS	
Brazil (Gomes et al., 2022)	*NR5A1*	chr9	c.77C>T	p.Gly26Val	LP	
	*DHX37*	chr12	c.1474G>C	p.Ala494Pro	VUS	
Brazil (Gomes et al., 2022)	*MAP3K1*	chr5	c.1628A>G	p.His543Arg	VUS	
	*GATA4*	chr8	c.644G>T	p.Arg215Ile	VUS	
Brazil (Gomes et al., 2022)	*FGFR2*	chr10	c.1358C>T	p.Ser453Leu	LP	
	*MAP3K1*	chr5	c.1916T>C	p.Leu639Pro	LP	
America (Arboleda et al., 2013)	*GATA4*	chr8	c..228G>A	p.V380M		46, XY gonadal dysgenesis
	*DMRT2*	chr9	c.194 G>A	p.R382Q		
	*CBX2*	chr17	c.228 C>T	p.A452V		
	*DMRT2*	chr9	c.100 C> G	p.P271A		
	*SRY*	chrY	c.225 T>C	p.Y127C		
America (Arboleda et al., 2013)	*AMHR2*	chr12	c.136 A>C	p.T108P		46, XY gonadal dysgenesis and galactosemia
	*ZFPM2*	chr8	c.228 G>C	p.S210T		
America (Arboleda et al., 2013)	*CBX2*	chr17	c.228 C>T	p.A452V		46, XX testicular DSD
	*S0X3*	chrX	c.228 C>T	p.A43T		
America (Arboleda et al., 2013)	*DMRT1*	chr9	c.46 T>G	p.V → G *b*		46, XY DSD severe combined adrenal and gonadal deficiency
	*CYP11A1*	chr15	c.208 ins A	Intronic splice-site mutation		
	*CYP11A1*	chr15	c.726 delT	Frameshift, early-termination		
America (Arboleda et al., 2013)	*BNC2*	chr9	c.2371T>C	p.Tyr791His	VUS	
	*FGFR1*	chr8	c.320C>T	p.Ser107Leu	VUS	
Algeria (Zidoune et al., 2022)	*DHH*	chr12	c.913G>A	p.G305R	VUS	Phallus < 1 cm, absence of gonads
	*GPRC6A*	chr6	c.2323dupT	p.Y775fs	B	
Algeria (Zidoune et al., 2022)	*ANOS1*	chrX	c.1187C>T	p.S396L	VUS	Genital bud (4 cm), posterior orifice, poorly developed, labioscrotal folds, absence of the left gonad and the right located in the abdomen
	*WT1*	chr11	c.913C>G	p.A100G	LB	
Algeria (Zidoune et al., 2022)	*NR5A1*	chr9	c.1223A>C	p.H408P	P	Micropenis (2 cm), 2 orifices, anogenital, distance of 4 cm, hypotrophic and inguinal testis,
	*MAP3K1*	chr5	c.3418A>G	p.M1140V	VUS	
	*CTU2*	chr16	c.710C>T	p.A237V	VUS	
Algeria (Zidoune et al., 2022)	*DHX37*	chr12	c.923G>A	p.R308Q	P	Micropenis (<0.5 cm), posterior hypospadia, hypoplastic labia, absence of gonads
	*GLI2*	chr16	c.1289C>G	p.A430G	VUS	
	*CCDC141*	chr2	c.3782C>T	p.A1261V	VUS	
Algeria (Zidoune et al., 2022)	*MYRF*	chr11	c.1222A>G	p.I408V	VUS	Genital bud (2 cm), perineal hypospadias, bifid scrotum, Uterus remnant present, uterovaginal cavity present, the right test located nguinal, absence of the left gonad
	*CCDC141*	chr2	c.1131G>T	p.K377N	VUS	
	*TGIF1*	chr18	c.239dupC	p.A80fs	VUS	
Algeria (Zidoune et al., 2022)	*DHX37*	chr12	c.923G>A	p.R308Q	P	Micropenis (0.5 cm), fused pigmented labia, fusion of labia minora, absence of gonads
	*SLC29A3*	chr10	c.971C>T	p.P324L	VUS	
	*CCDC141*	chr2	c.1979G>A	p.R660Q	VUS	
Algeria (Zidoune et al., 2022)	*FGFR2*	chr10	c.1132A>G	p.I378V	VUS	Clitoromegaly (1.5 cm), bifid poorly developed labiscrotaln folds, presence of a left structure correspond probably to a Müllerian Ducts, inguinal testis
	*FANCD2*	chr3	c.2965C>G	p.P989A	VUS	
Algeria (Zidoune et al., 2022)	*MAP3K1*	chr5	c.3557A>G	p.E1186G	VUS	Micropenis (1 cm), phimosis, urethral orifice, developed scrotum, the right testis located in the scrotum and the left missing
	*MYRF*	chr11	c.1222A>G	p.I408V	VUS	
Algeria (Zidoune et al., 2022)	*SOX8*	chr16	c.1264G>A	p.G422S	VUS	Penis (5 cm), urogenital sinus closed, urethral orifice, developed scrotum, atrophic prostate present, absence of gonads
	*PROKR2*	chr20	c.868C>T	p.P290S	VUS	
	*PLXNA3*	chrX	c.787_796del	p.V263fs	VUS	
	*FLNA*	chr X	c.2449C>T	p.P817S	VUS	
	*NIPBL*	chr5	c.6954+3A>G	—	VUS	
	*SLC29A3*	chr10	c.325G>A	p.V109I	VUS	
	*GLI3*	chr7	c.1527G>C	p.E509D	VUS	
Algeria (Zidoune et al., 2022)	*AMH*	chr19	c.127_128del	p.L43fs	P	Developed phallus, scrotum, Müllerian Ducts present, testes located in the abdominal cavity, the right one: testicularparenchyma, seminiferous tubules with immature
	*GPRC6A*	chr6	c.1969T>C	p.F657L	LB	
The United Kingdom (Hughes et al., 2019)	*CYP11A1*	chr15	c.989C>T	p.Thr330Met	VUS	? CYP11A1 imbalance
	*MAMLDI*	chr X	c.2009C>T	p.Thr670Ile	VUS	
The United Kingdom (Hughes et al., 2019)	*CBX2*	chr17	c.1411C>G	p.Pro471Ala	VUS	Tall stature, uterus present, no obvious ovaries
	*AMH*	chr19	c.53C>T	p.Ala18Val	VUS	
		chr19	c.1556C>T	p.Ala519Val	VUS	
The United Kingdom (Hughes et al., 2019)	*CYP11A1*	chr15	c.940G>A	p.Glu314Lys	VUS	Hypospadias and penoscrotal transposition
	*HSD17B3*	chr9	c.133C>T	p.Arg45Trp	VUS	
	*HSD17B3 (Allele 2)*	chr9	c.133C>T	p.Arg45Trp	VUS	
The United Kingdom (Hughes et al., 2019)	*LHCGR*	chr2	c.828delC	p.Ser277Alafs*32	P	?46, XY DSD
	*CBX2*	chr17	c.785G>A	p.Arg262Gln	VUS	
The United Kingdom (Hughes et al., 2019)	*LHCGR*	chr2	c.458+3A>GV			Ambiguous genitalia, complete labial fusion
	*NR5A1*	chr9	c.486C>T	p.=		
The United Kingdom (Hughes et al., 2019)	*ATRX*	chrX	c.2595C>G	p.(His865Gln)V		?46, XY DSD
	*AMH*	chr19	c.-2C>TV			
The United Kingdom (Hughes et al., 2019)	*AMH*	chr19	c.35T>G	p.(Val12Gly)P		hypospadias
	*AMH*	chr19	c.74C>GV			
	*CBX2*	chr17	c.565G>A	p.(Ala189Thr)V		
Ukraine (Globa et al., 2022)	*NR5A1*	chr9		p.G35D	P	Clitoromegaly and urogenital sinus. Right-sided inguinal hernia at 3 years. Migratory gonads: from abdominal position to the inguinal canals
	*ABCD1*	chrX		p.M539V	LP	
Ukraine (Globa et al., 2022)	*CACNA1A*	chr19		p.I219V	P	Bilateral cryptorchidism, scrotum hypospadias, micropenia, abdominal, cavity
	*ESR2*	chr14		p.Y49C	LP	
Ukraine (Globa et al., 2022)	*WDR34*	chr9		p.Q158X	VUS	46, XY, U, AG
	*WDR35*	chr2		p.T1020R	VUS	
	*DYNC2H1*	chr11		p.S2281C	VUS	
	*CDT1*	chr16		p.R138W	VUS	
	*TRAF3IP1*	chr2		p.I386V	VUS	
	*SOX4*	chr6		p.A275V	VUS	
Ukraine (Globa et al., 2022)	*WDR11*	chr11		p.F1150L/p.V356I	VUS	46, XY, U, AG
	*MYRF*	chr11		p.A315V	VUS	
	*ANOS1*	chrX	c.856+3C>T		VUS	
	*CCDC141*	chr2		p.D767N	VUS	
Ukraine (Globa et al., 2022)	*HESX1*	chr3		p.V129I	VUS	46, XY, U
	*POU1F1*	chr3		p.D10G	VUS	
Ukraine (Globa et al., 2022)	*POU1F1*	chr3		p.D10G	VUS	46, XY, U
	*GNRHR*	chr4		p.P146S	VUS	
Ukraine (Globa et al., 2022)	*SAMD9*	chr7		p.R1040C	VUS	46, XY, TRS, severe immunodeficiency
	*STAT1*	chr2	c.1632+6G>A		VUS	
	*IKBKB*	chr8		p.A755S	VUS	
	*TAP2*	chr6		p.L59delinsLKLRGLL	VUS	
Ukraine (Globa et al., 2022)	*HSD3B2*	chr1		p.A167V	VUS	46, XY, GD (gonadoblastoma)
	*FANCM*	chr14		p.Ser497fs	VUS	
Ukraine (Globa et al., 2022)	*PTCH1*	chr9		p.T627M	VUS	46, XY, U, AG
	*LZTR1*	chr22		p.M695I	VUS	
Ukraine (Globa et al., 2022)	*LHCGR*	chr2		p.Y113N	VUS	46, XY, CAIS
	*LHX3*	chr9		p.A3V	VUS	
	*GNAS*	chr20		p.A426P	VUS	
	*PAX4*	chr7		p.C282R	VUS	
Ukraine (Globa et al., 2022)	*CHD7*	chr8		p.M2527L	VUS	46, XY, TRS
	*ANOS1*	chrX	c.256-2A>T		VUS	
Ukraine (Globa et al., 2022)	*WDR11*	chr10		p.A435T	VUS	46, XY, TRS
	*CHD7*	chr8		p.M340V	VUS	
	*POR*	chr7		p.V348I	VUS	
America (Globa et al., 2022)	*BNC2*	chr9	c.237T>C	p.Tyr791His	VUS	2-cm phallus, penoscrotal hypospadias, penoscrotal transposition, micropenis, chordee, bilateral descended testes
	*FGFR1*	chr8	c.320C>T	p.Ser107Leu	VUS	

^a^ The blank section was not stated in detail in the study cohorts.

Two or more DSD-associated genes mutations present in patients in each country studied are shown in Table 2.

### Oceania

#### Australia

In 2016, MPS (including 1,031 genes) was used in Australia to conduct a large cohort study of DSDs. A total of 278 patients with 46, XY DSDs and 48 patients with 46, XX DSDs were enrolled after excluding patients with CAH and sex chromosome abnormalities. The cohort covered 12 countries, and the researchers divided patients into Asia, Australia/New Zealand, and Europe. This study showed that the proportion of patients with DSD variants was similar in different regions, and the diagnosis rate varied (from 33% in Asia [58 of 174 patients] to 45% in Australia/New Zealand [41 of 90 patients]) in different regions. In the 46, XY DSD cohort, genetic diagnosis was possible in 40% of patients with gonadal dysplasia and 60% of patients with androgen synthesis and dysplasia. A total of 43% of 46, XY DSD patients received a possible genetic diagnosis. Variations in the *AR* (17.22%, 26/151) gene were the most common, followed by *NR5A1* (10.60%, 16/151) and *SR5A2* (8.61%, 13/151) (Eggers et al., 2016).

## Summary of worldwide 46, XY DSD cohorts

In the above-collected studies, the molecular diagnostic rate for patients with 46, XY DSD ranged from a low of 24.3% to a high of 64.3%, and the most common mutated genes were *AR*, *NR5A1*, and *SRD5A2*. We found that patients with androgen synthesis disorders accounted for the greatest percentage of all patients. These three genes, which encode key enzymes for the androgen receptor, steroid nuclear receptor, and dihydrotestosterone, are involved in the process of androgen synthesis and action (Gulía et al., 2018; Dhurat et al., 2020; Morohashi et al., 2020). The gene encoding the androgen receptor (*AR*), located on the X chromosome, causes AIS (Gulía et al., 2018). *NR5A1* is a transcription factor of the steroidogenic nuclear receptor superfamily that regulates the transcription of many reproductive, steroidogenesis, and male sexual differentiation genes (Morohashi et al., 2020). Steroid 5-alpha-reductase catalyzes the conversion of testosterone to the more potent dihydrotestosterone, the lack of which often leads to the development of micropenis (Dhurat et al., 2020).

The results of the individual cohorts were influenced by the inclusion criteria. There are no mutations with a clear geographic or ethnic predominance. Patients with multiple mutations tended to have more severe clinical manifestations. Conversely, patients with milder manifestations tended to have difficulty obtaining a molecular diagnosis. The diagnostic rates for these cohort studies may be correlated with the number of patients and the incidence of disease, as well as coincidence. The number of genes included in gene panels is limited, and WES can only detect coding regions. Therefore, WGS might gradually become the main modality used to detect such diseases, which can expand the genetic spectrum of human disease-causing genes. There are still many genes for which the association with DSDs is unclear, and some patients also lack a definitive molecular diagnosis. The basic information on 55 patients with multiple mutations is listed in Table 2; in total, 38 patients had 2 DSD-related mutations, 10 had 3 DSD-related mutations, and 7 had 4 or more DSD-related mutations. For 46, XY DSDs, there is no clear genotype–phenotype correlation, and the phenotypic variations may be due to other genomic variants or environmental influences on regulatory pathways required for gonad formation and/or function. Therefore, in patients with multiple mutations, it is necessary to determine whether the simultaneous mutation of these genes causes the disease or whether only one of the mutations is associated with the disease.

The results of polygenic testing suggest the diversity of disease etiologies and the complexity of their manifestations and, at the same time, provide direction for diagnosis. Patients with oligogenic mutations do not develop more severe phenotypes. The severity of the manifestation is more likely to be the result of the action of the mutated gene and the altered protein.

### Updated monogenic forms of 46, XY DSDs

A monogenic phenotype results from the action of a pair of genes or alleles. The gene variants described here are considered monogenic causes of DSDs (i.e., variants in these genes trigger DSDs, and DSDs would not occur without these variants). The etiology of 46, XY DSDs is often categorized as androgen synthesis or action disorders, AMH synthesis or action disorders, and 46, XY GD.

The genes involved in hormone synthesis and action are *AR*, *SRD5A2*, *HSD17B3*, *DHCR7*, *LHCGR*, *STAR*, *CYP11A1*, *CYP17A1*, *CYB5A*, *HSD3B2*, *AMH*, *AMHR2*, *AKR1C2*, and *AKR1C4* (Zhang et al., 2023). In the early stages of research into DSDs, most studies favored factors related to hormone synthesis or action, and *AR* was considered the most likely gene to cause the disease. In the included cohort studies, *AR* was the most common causative gene for 46, XY DSDs. However, with the continuous development of sequencing technology and improvements in basic experiments, new genes that have not been previously linked to this disease’, such as *CBX2*, *DHX37*, *GATA4*, *DHH*, and other genes in the *SOX* gene family, are being identified.

**FIGURE 1:**
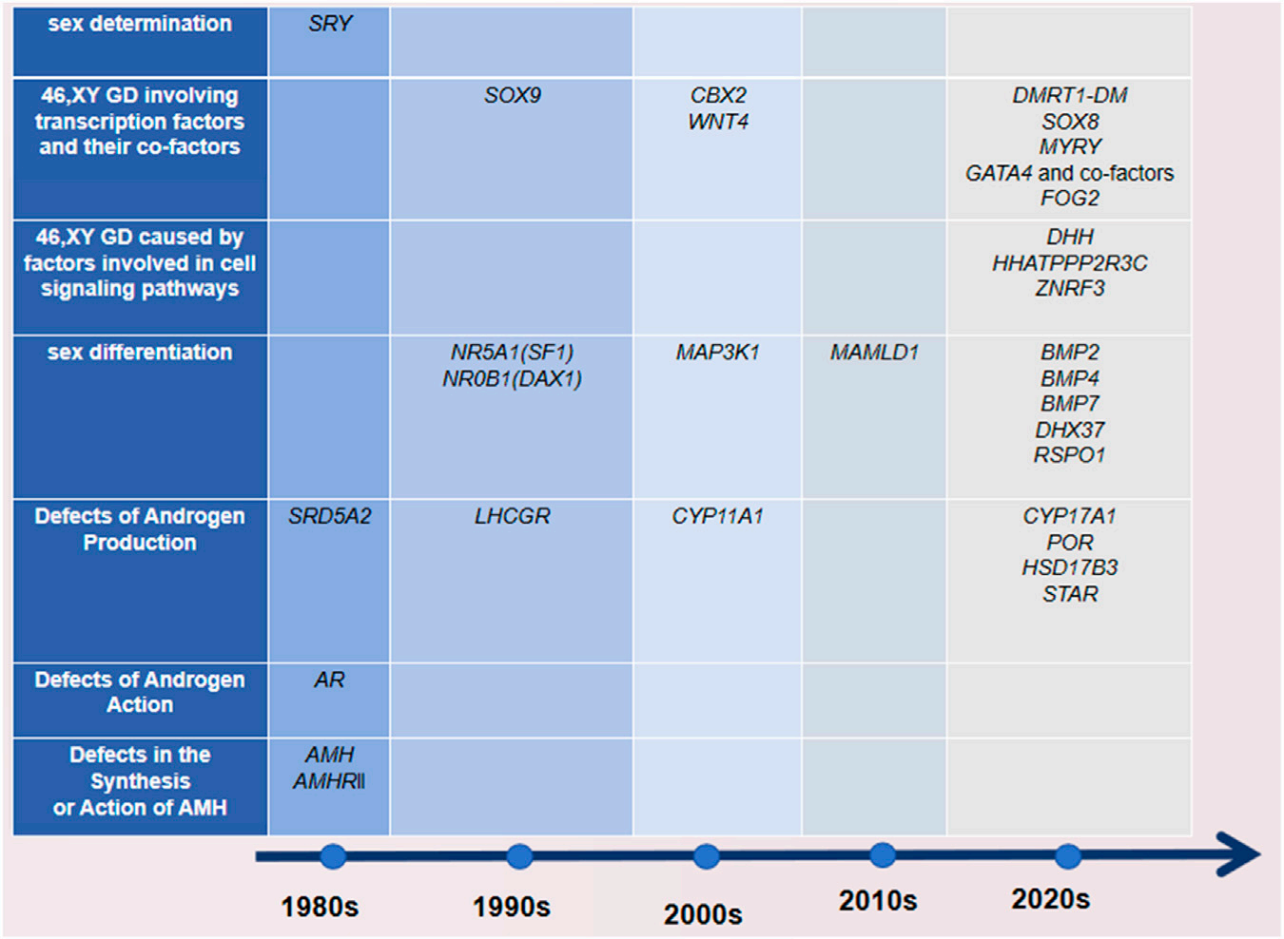
Time of discovery and classification of DSD-related genes. The time standard found here refers to when there are basic experiments, and it is detected in the molecular assay of the patients.

We have realized the updated monogenic mutations involved in the gonadal decision and differentiation phases leading to GD occurrence, including *CBX2*, *DMRT1*, *GATA4* and *2FPM2 (FOG2)*, *MYRF*, the SOX gene family, *DHX37*, *DHH* and *HHAT*, *PPP2R3C*, and *ZNRF3* (Elzaiat et al., 2022). Figure 1 shows that with the continuous updating of testing technology, more genes related to DSDs have been found to have diagnostic significance.

Next, we briefly discuss the genes recently proposed to be responsible for monogenic 46, XY GD in these cohorts. Current data suggest that in the testicular developmental pathway, *CBX2* stabilizes the testis pathway by blocking the upregulated expression of genes in the ovarian pathway (Garcia-Moreno et al., 2019). We found a mutation in *CBX2* in patients with polygenic mutations (c.228 C > T (p.Ala452Val), c.1411C > G (p.Pro471Ala), c.785G > A (p.Arg262Gln), and c.565G > A (p.Ala189Thr)). The two *CBX2* variants found in the United States had the same locus, c.228C > T (p.A452V).

*DHX37* is a member of the DEXH family of RNA helicases, and *DHX37* mutations have been previously shown to cause 46, XY GD, testicular regression sequence (TRS), or anencephaly. The cause of these testicular developmental disorders has been identified as effects on β-conjugated proteins (Wan et al., 2023). The following mutation sites were found in the molecular assay results from two Algerian polygenic patients: c.2209G>A (p.Ala737Thr), c.1474G>C (p.Ala494Pro), and c.923G>A (p.Arg308Gln).

*GATA4* is a zinc finger (ZF) transcription factor characterized by the presence of two conserved type IV ZF structural domains (amino acids 217-241 and 271-295), which interact with *NR5A1* to regulate gene expression during testis assay and differentiation (Robert et al., 2006). Similarly, we found *GATA4* c.644G>T (p.Arg215Ile) mutations in two patients in Brazil.

## Conclusion

*AR*, *NR5A1*, and *SRD5A2* are the most common mutated genes because of differences in the labeling of the cohorts in different countries, especially China, Brazil, and Australia. The mutation frequency of the *AR* gene in these cohorts ranged from 4.05% to 55.56%; the highest mutation frequency of *NR5A1* was 22.22% and that of *SRD5A2* was 11.11%, which were affected by the number of patients in the cohorts.

Among the known genes associated with nonsyndromic 46, XY gonadal agenesis, variants of *SRY*, *NR5A1*, and *MAP3K1* are the most common (Reyes et al., 2023). The *MAP3K1* mutations detected in the United States, Australia, and South Korea cohorts were completely different. The variants detected in the United States were p. Ly616Arg, p. Rg339Gln, p. Ly616Arg, and p. RO257Leu (Baxter et al., 2015a). In Australia, the *MAP3K1* variants were p.Leu189Arg, p.Met312Leu, p.Ala1443Val (Eggers et al., 2016), and in Korea, the main variant was p. E1293K (Kim et al., 2017). For the international molecular diagnosis of DSDs, the limited ethnic or geographic differences we found may result from either the lack of reference to the patient’s ethnic origin in the article or an insufficient number of studies. The results of molecular detection were affected by the inclusion criteria and the detection method.

Multiple mutations have been identified in national studies, although the mutated genes were not commonly thought to be associated with DSDs. However, there is no clear relationship between the number of mutations in a gene and the severity of the disease; rather, the severity of the disease is directly related to the role of the mutated gene in sexual differentiation and development. Mutations in two key genes can lead to more severe manifestations. This also suggests that DSD is not a monogenic disease. This study also provides direction for the molecular diagnosis of DSD patients. Mutations in multiple genes may not only lead to the development of one disease but also to multisystemic lesions. Therefore, it is important to observe and follow patients with multiple abnormal manifestations in multiple systems in the clinic. Obtaining a definitive molecular diagnosis is important for understanding not only the cause of the disease but also the prognosis of the patient, as well as providing targets for gene therapy and even aiding in genetic counseling. The history of the discovery of disease-causing genes shows that an increasing number of disease-causing genes are discovered with the advancement of techniques and technology. Even with the current widespread use of WES, the cause of the disease in nearly half of 46, XY DSD patients still cannot be clearly identified.

The timing of diagnosis is critical for patients with 46, XY DSDs, some of whom may not ever receive a definitive diagnosis. Treatment is a challenge in terms of gender rearing as most of these patients reach puberty and are clarifying their gender identity as well as their sexual orientation, and we need to account for the thoughts of the patients and their families. In the absence of curative treatments, prenatal diagnosis is crucial for DSDs and other rare diseases. Patients with poor gonadal differentiation have an increased risk of germ cell tumors, and gonadoblastoma is the most common in this patient group (Reyes et al., 2023).

The molecular diagnosis of 46, XY DSDs remains a substantial challenge, and with the development and innovation of molecular techniques, the diagnosis of these patients can be greatly improved in the future. With the widespread use of WGS for rare diseases, it may be a better test for the diagnosis of DSDs because the whole genome is sequenced rather than only protein-coding regions.
